# SACCHARIS: an automated pipeline to streamline discovery of carbohydrate active enzyme activities within polyspecific families and de novo sequence datasets

**DOI:** 10.1186/s13068-018-1027-x

**Published:** 2018-02-05

**Authors:** Darryl R. Jones, Dallas Thomas, Nicholas Alger, Ata Ghavidel, G. Douglas Inglis, D. Wade Abbott

**Affiliations:** Lethbridge Research and Development Centre, Agriculture and Agri-Food Canada, 5403-1st Avenue South, Lethbridge, AB T1J 4B1 Canada

**Keywords:** Carbohydrate active enzyme, Carbohydrate, Phylogeny, Enzyme discovery, Bioprocessing

## Abstract

**Background:**

Deposition of new genetic sequences in online databases is expanding at an unprecedented rate. As a result, sequence identification continues to outpace functional characterization of carbohydrate active enzymes (CAZymes). In this paradigm, the discovery of enzymes with novel functions is often hindered by high volumes of uncharacterized sequences particularly when the enzyme sequence belongs to a family that exhibits diverse functional specificities (i.e., polyspecificity). Therefore, to direct sequence-based discovery and characterization of new enzyme activities we have developed an automated in silico pipeline entitled: Sequence Analysis and Clustering of CarboHydrate Active enzymes for Rapid Informed prediction of Specificity (SACCHARIS). This pipeline streamlines the selection of uncharacterized sequences for discovery of new CAZyme or CBM specificity from families currently maintained on the CAZy website or within user-defined datasets.

**Results:**

SACCHARIS was used to generate a phylogenetic tree of a GH43, a CAZyme family with defined subfamily designations. This analysis confirmed that large datasets can be organized into sequence clusters of manageable sizes that possess related functions. Seeding this tree with a GH43 sequence from *Bacteroides dorei* DSM 17855 (BdGH43b, revealed it partitioned as a single sequence within the tree. This pattern was consistent with it possessing a unique enzyme activity for GH43 as BdGH43b is the first described α-glucanase described for this family. The capacity of SACCHARIS to extract and cluster characterized carbohydrate binding module sequences was demonstrated using family 6 CBMs (i.e., CBM6s). This CBM family displays a polyspecific ligand binding profile and contains many structurally determined members. Using SACCHARIS to identify a cluster of divergent sequences, a CBM6 sequence from a unique clade was demonstrated to bind yeast mannan, which represents the first description of an α-mannan binding CBM. Additionally, we have performed a CAZome analysis of an in-house sequenced bacterial genome and a comparative analysis of *B. thetaiotaomicron* VPI-5482 and *B. thetaiotaomicron* 7330, to demonstrate that SACCHARIS can generate “CAZome fingerprints”, which differentiate between the saccharolytic potential of two related strains in silico.

**Conclusions:**

Establishing sequence-function and sequence-structure relationships in polyspecific CAZyme families are promising approaches for streamlining enzyme discovery. SACCHARIS facilitates this process by embedding CAZyme and CBM family trees generated from biochemically to structurally characterized sequences, with protein sequences that have unknown functions. In addition, these trees can be integrated with user-defined datasets (e.g., genomics, metagenomics, and transcriptomics) to inform experimental characterization of new CAZymes or CBMs not currently curated, and for researchers to compare differential sequence patterns between entire CAZomes. In this light, SACCHARIS provides an in silico tool that can be tailored for enzyme bioprospecting in datasets of increasing complexity and for diverse applications in glycobiotechnology.

## Background

The Carbohydrate Active Enzyme (CAZyme) database (ca. 1998) is an online repository that curates enzyme sequences predicted or known to be involved in the metabolism of carbohydrates [1]. CAZymes are classified into five different enzyme classes, including glycoside hydrolases (GHs), polysaccharide lyases (PLs), auxiliary activities (AAs), carbohydrate esterases (CEs), and glycosyl transferases (GTs). GHs [2] and PLs [3] cleave glycosidic linkages by a hydrolytic and β-elimination mechanism, respectively. AAs are a recently defined class of oxidative enzymes that depolymerize crystalline polysaccharides, such as cellulose, chitin, starch, and lignin [4]. CEs hydrolyze O- and N-linked of carbohydrate esters generating alcohol and acid products [5]. GTs catalyze biosynthetic reactions and are involved in the glycosylation of acceptor molecules, such as carbohydrates, lipids and proteins [6]. In addition to these five classes of enzymes, carbohydrate binding modules (i.e., CBMs) are also curated within the CAZy database. CBMs are non-catalytic sequences that fold into independent functional units; CBMs potentiate appended enzyme activity by targeting and/or concentrating effects [7, 8].

The CAZy database is an indispensable resource for guiding the discovery and characterization of CAZymes important for diverse aspects of glycobiotechnology, including agriculture, human health, and bioconversion of renewable resources for bioproducts and biofuels [9–11]. Due to the increased accessibility and affordability of next-generation sequencing technologies, genetic sequence space deposited into online databases is expanding at an unprecedented rate and continues to outpace the functional characterization of CAZymes. For example, GH family 43 (GH43), a family known to be active on diverse arabinosyl- and xylosyl-configured substrates [12–14] abundant within non-cellulosic plant cell wall polysaccharides, only had 1.9% of its > 7000 total entries functionally characterized at the time of this analysis. Similarly, GH family 92 (GH92), a family with diverse activities on α-mannosyl substrates (e.g., α-1,2; α-1,3; α-1,4; and α-1,6 mannosides) [15] found in feedstocks generated from distillation residues (e.g., dried distillers grains with solubles), only had 1.2% of its > 2400 sequences characterized. This pattern also extends to CBMs. CBM6 is a polyspecific family with diverse plant and algal cell wall carbohydrate binding specificities that can possess two distinct binding sites: variable loop site (VLS) and concave face site (CFS) [16]. At the time of this analysis, 5.1% of 1922 sequences containing a CBM6 were associated with a characterized enzyme. This estimate of characterized CBM6 specificity is likely overrepresented, however, as known activities of CAZymes associated with a CBM6 do not necessarily equate to an accurate representation of CBM6 binding specificity [17]. Despite these reports, the full pallet of substrate specificity and/or modes of action (e.g., exo-acting versus endo-acting; distributive versus processive) within GH43 and GH92, and ligand binding specificities within CBM6 likely remain to be discovered. In this light, uncharacterized sequences from CAZyme families with polyspecific profiles represent untapped repositories for enzyme discovery. Recent bioprospecting within known families has resulted in the discovery of novel activities and the generation of valuable biocatalysts [18–21].

Often enzyme discovery within polyspecific families is hindered by large sequence volume and inherent multimodularity within some CAZyme families, which makes alignments difficult. To help streamline the characterization of CAZyme function, several bioinformatic approaches have been developed. These include subfamily delineation within a defined group of GH families [20, 22–24]; PULDB (Polysaccharide Utilization Loci DataBase; [25]), an online tool associated with the CAZy website which provides comparative predictions of enzyme activities within *Bacteroides* spp. catabolic pathways active on defined substrates; dbCAN (database for automated Carbohydrate-active enzyme ANnotation [26]), a web server that identifies potential CAZymes within uncharacterized sequences; and ancestral sequence reconstruction [27], which calculates the most likely progenitor sequence between two related sequences and can be used to map the evolution of contemporary and ancestral enzyme activities [28] or binding specificities [29]. Despite these advances, an automated method for the rapid identification of uncharacterized CAZyme sequence space with capacity to handle large datasets and target user-defined CAZyme function is currently lacking.

**Fig. 1:**
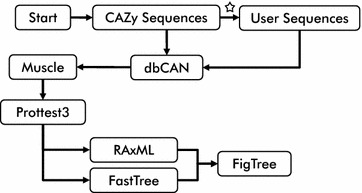
Flow diagram of SACCHARIS. The pipeline initiates with a query of http://www.cazy.org and outputs protein sequence. User-generated protein sequences from sequence datasets can be added (white star). dbCAN [26] is utilized for identification of modular boundaries. Selected enzyme sequences are extracted and boundaries pruned using in-house tools. Alignment is performed via MUSCLE [54], and phylogenetic grouping by RAxML [57] or FAstTree [56], respectively. ProtTest [55] is used for the selection of the best-fit model. Trees are outputted as Newick files for tree plotting in by FigTree

Presented here is a pipeline entitled: Sequence Analysis and Clustering of CarboHydrate Active enzymes for Rapid Informed prediction of Specificity (SACCHARIS; from the Greek “sákkʰaris” or “sugar”) (Fig. 1). SACCHARIS enables the user to extract entire sequence lists from a designated CAZyme family, en bloc trim multimodular enzymes to their modular boundaries, align the trimmed sequences, and display statistically derived phylogenies with vector graphics suitable for publication. The outputs from this pipeline provide direct and easy-to-interpret insights into new functional space within a CAZyme or CBM family. In addition to comprehensive family analyses, extractions can be specified for taxonomic divisions, biochemically characterized sequences, or structurally characterized sequences. Additionally, by embedding experimentally characterized sequences into user-generated datasets (e.g., genomic, metagenomic, and transcriptomic) this pipeline can streamline enzyme discovery for diverse research applications.

In this study, GH43 and CBM6 have been used to generate sequence-based clades. This phylogenetic analyses led to the discovery of a novel enzyme and binding specificity, respectively. Firstly, a GH43 from *Bacteroides dorei* DSM 17855 (BdGH43b) was identified as being an endo-acting enzyme that cleaves α-glucans, which varies from all other previously described GH43s that are reported to act on β-d- or α-l-configured substrates. Additionally, a CBM6 from *Cellulosimicrobium cellulans* (CcCBM6a) was demonstrated to bind yeast mannan, which represents the first α-mannan binding CBM described in the literature. Importantly, SACCHARIS analysis of individual families can be extended to entire genomes. Such analyses, which we refer to here as ‘CAZome fingerprinting’, provide in silico metabolic snapshots that can be used to predict saccharolytic potential with higher resolution. As a proof of principle, we have characterized the CAZome fingerprints of *Campylobacter jejuni* subsp. jejuni NCTC 11168-GSv, a genome previously sequenced by our group [30], and the differential CAZome fingerprints of two closely related strains of *Bacteroides thetaiotaomicron*. We anticipate that the SACCHARIS pipeline will be of interest to the glycobiotechnology community as it can be used to generate informative phylogenies for any enzyme or CBM family currently maintained on the CAZy website, provide differential CAZome analysis of genomes, and perhaps most importantly, be harnessed to bioprospect enzymes within user-defined meta-datasets.

## Results and discussion

### SACCHARIS generates accurate phylogenetic trees

**Fig. 2:**
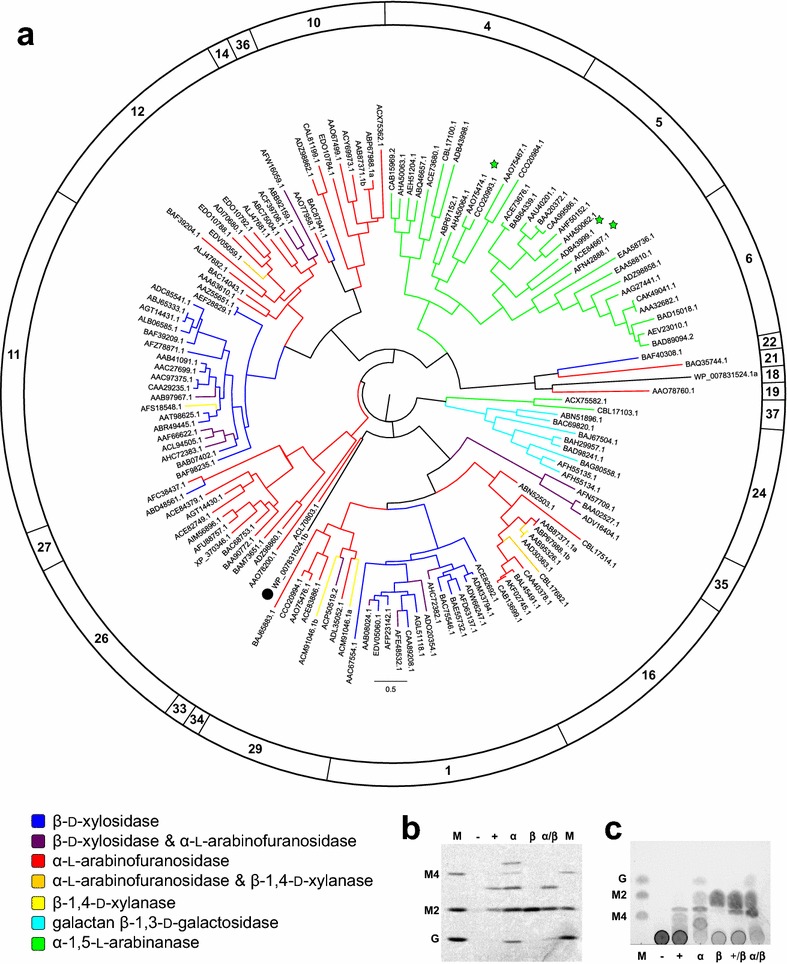
Phylogenetic tree using characterized sequences from GH43. **a** Subfamily assignments defined by http://www.CAZy.org [20] are shown in the outer circle, and members of the tree are coloured based on their characterized activity. Green stars indicate the distribution of exo-acting arabinanases. BdGH43b is indicated with a black circle. BdGH43b endo-α-glucosidase activity; **b** Fluorescent assisted carbohydrate electrophoresis and **c** thin-layer chromatography of products generated by BdGH43b (+) following in vitro digestion of soluble potato starch. Control digestions of starch were performed with endo-α-amylase (α), maltose releasing β-amylase (β), and no enzyme (−). Marker (M) containing; glucose (G), maltobiose (M2), maltotetrose (M4) as standards are indicated

To evaluate the accuracy of SACCHARIS (Fig. 1) a tree of characterized GH43s embedded with two uncharacterized GH43s from *B. dorei* (BdGH43a and BdGH43b) was constructed (Fig. 2a). GH43 is a polyspecific family that is active on non-cellulosic plant cell wall polysaccharides. This family has been divided into thirty-seven defined subfamilies [20] (i.e., GH43_1 to GH43_37) with biochemically determined activities that include α-l-arabinofuranosidase (EC 3.2.1.55), β-d-xylosidase (EC 3.2.1.37), α-1,5-l-arabinanase (EC 3.2.1.99), β-1,4-d-xylanase (EC 3.2.1.8), and galactan β-1,3-d-galactosidase (EC 3.2.1.145) [1]. Trees generated with ‘characterized’ GH43 sequences using SACCHARIS produces twenty distinct clades, plus BdGH43a and BdGH43b, which is consistent with the twenty subfamilies with characterized functions defined in CAZy at the time of this analysis [1, 20] (Fig. 2a).

The majority of characterized GH43s have been identified as α-l-arabinofuranosidases; these activities are distributed into five main clusters in the SACCHARIS phylogeny encompassing fourteen identified subfamilies (GH43_1, GH43_10–12, GH43_14, GH43_16, GH43_19, GH43_21, GH43_26, GH43_27, GH43_29, GH43_33, GH43_35, and GH43_36). The second most abundant described activity within GH43 is β-1,4-d-xylosidase, which is found within ten subfamilies (GH43_1, GH43_11–12, GH43_14, GH43_16, GH43_22, GH43_27, GH43_29, GH43_35, and GH43_36); the major clusters are primarily represented by subfamilies GH43_1 and GH43_11. The prevalence of reported activities for arabinofuranosidases and xylosidases likely results from the availability of small synthetic arabinose and xylose substrates for rapid identification of activity; the characterization of more complex substrate specificities depend on the synthesis or purification of appropriate substrates. Arabinofuranosidase and xylosidase activity are not necessarily mutually exclusive. GH43s with dual function are distributed amongst seven clades within the phylogeny, and GH43_35 is comprised entirely of members with dual activity [31–33]. Such cross-specificity may result from similar stereochemical configuration of C1, C2, and C3, in α-l-arabinofuranose and β-d-xylopyranose [20].

The α-1,5-l-arabinanases are solely distributed among two clusters within the phylogenetic tree that span four subfamilies (GH43_4–6, and GH43_37). The majority of identified arabinanases are endo-acting; however, three exo-α-1,5-l-arabinanases, ACE84667.1, ADB43999.1, and CCO20984.1 have also been described (Fig. 2a). The former two are closely related enzymes that partition into GH43_5 [20]. CCO20984.1 was discovered from fungus-growing *Pseudacanthotermes militaris* termite gut and belongs to subfamily GH43_4 [34]. The molecular basis for exo-activity in this enzyme is unclear as it is closely related to the endo-arabinanase AAO75474.1 from the intestinal symbiont *Bacteroides thetaiotaomicron* VPI-5482.

Rare activities within GH43 include the galactan β-1,3-galactosidases, which are limited to a single subfamily (GH43_24), and the β-1,4-d-xylanases (GH43_11, GH43_12, GH43_16, and GH43_29). The galactan β-1,3-galactosidases display an altered catalytic triad configuration to other GH43s [35]. In contrast, the xylanases from family 43 are more distributed throughout the phylogenetic tree, and can be found within three clades. Three of these enzymes (AAD30363.1, CBL17682.1, and EDV05059.1) possess both xylanase and arabinofuranosidase activity [1, 36].

To investigate the ability of SACCHARIS to streamline enzyme discovery, we have investigated the sequence-function relationship of BdGH43b (WP_007831524.1b). BdGH43b was selected for embedding into the GH43 tree because it had an unusual bimodal architecture with tandem GH43s (BdGH43a and BdGH43b) that are classified into subfamily 18 and 34, respectively. Additionally, BdGH43b diverges early from the GH43_33 subfamily, which contains a single entry from *Halothermothrix orenii* H 168 that is classified as an α-l-arabinofuranosidase (HoGH43; ACL70803.1). The structure of HoGH43 has been determined, and was noted to have a structurally unique active site [37]. To explore the activity of BdGH43b, reactions against common GH43 substrates were performed. BdGH43b did not display *bona fide* activity on PNP-α-l-arabinopyranoside, PNP-β-d-xylopyranoside, PNP-β-d-glucopyranoside, PNP-α-l-arabinofuranoside and PNP-β-d-galactopyranoside. Additionally, there was no activity detected on α-l-arabinan or β-d-xylan. Following this BdGH43b was screened against a panel of other substrates, including galactans, pectins, and α-glucans, which revealed that it released maltooligosaccharides from starch (Fig. 2b, c). This represents a unique activity for GH43 and the first enzyme from this family active on α-linked d-glucans.

### Delineation of structurally and functionally characterized CBMs using SACCHARIS

**Fig. 3:**
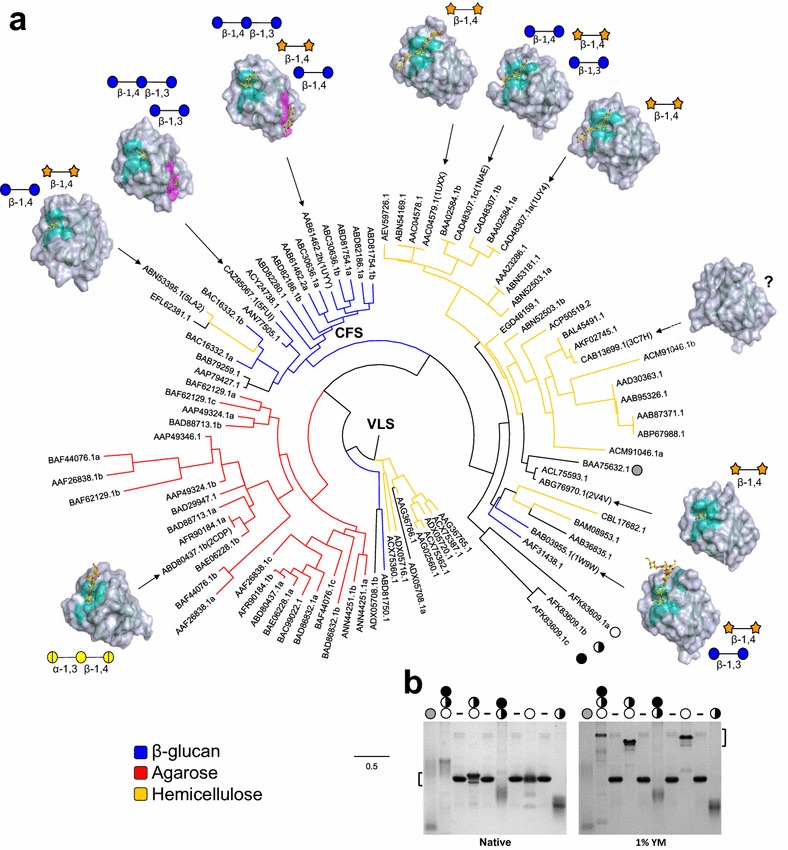
Distribution of CBM6 structures within a tree of CBM6s associated with characterized CAZymes. **a** Phylogenetic tree of characterized CBM6s (*n* = 90) were plotted with SACCHARIS. CBM6s with known three-dimensional structures where then mapped onto the tree and are indicated by their PDB ID. Rendered surface models with their bound ligands shown as yellow sticks are shown. For each structure the residues comprising the VLS are displayed in cyan and those of the CFS in magenta. Schematic representations of the sugar and stereochemical linkage recognized by each CBM are also displayed (blue circle = glucose, orange star = xylose, yellow circle = galactose, hatched yellow circle = 3,6-anhydro-l-galactose). Members of the tree are coloured based on the substrate that the appended catalytic module is active on. The black and white circles represent the CBM6s synthesized and tested for binding: BbCBM6 (grey circle), CcCBM6a (white circle), CcCBM6b (hatched circle), and CcCBM6c (black circle). **b** Affinity gel of various constructs of BbCBM6 and CcCBM6a–c. BSA controls are indicated with a dash. Equal amounts of CBM6s were run in acrylamide gels in absence (Native) and presence of 1% yeast mannan (YM)

The coverage of functionally or structurally characterized proteins within a CAZyme or CBM family is often difficult to ascertain without performing a complete phylogenetic tree. Although subfamily delineation helps in this regard, subfamilies have only been defined for a limited number of CAZyme families [1, 20, 22–24]. Within families (and clans) structural folds are conserved, but without detailed knowledge of CAZyme or CBM specificities these structures will likely not be informative for revealing function of uncharacterized members as subtle changes in primary structure can lead to diverse specificities [17, 38, 39]. For example, CBM6 is a polyspecific CBM family that has been described to interact with diverse ligands, including: β-1,4-xylosyl-; β-1,3- and β-1,4-glucosyl-configured oligosaccharides; and the algal polysaccharides laminarin and agarose [17, 40–43]. CBM6s have two potential locations for binding sites (VLS and CFS; [16, 44]) and they can bind ligands through different mechanisms: endo-like CBMs (Type B) and exo-like CBMs (Type C) [7, 8]. Therefore, to explore the capacity of SACCHARIS to generate informative trees using structural and functional information from an established polyspecific CBM family, we have performed a SACCHARIS analysis using entries for “characterized” CBM6s [45] (Fig. 3). Ninety CBM6 sequences were extracted and plotted, and their distribution agrees with previous phylogenetic analysis [17, 46]. This family clusters into four main clades that reflect the specificity of their appended catalytic fragment. These include hemicellulose, xylan, β-glucans with a variety of linkages, and agarose.

Advances in the structural analysis of CBM6s have provided further insight into the evolution of function and the bi-functional binding sites. The majority of characterised CBM6s have been confirmed as VLS binders, and correspondingly, VLS CBM6s are widely distributed throughout the tree. CBM6 from *Bacillus subtilis* (PDB: 3C7H; [47]) is the sole exception as this CBM does not have a known binding function. The two characterized structures with both VLS and CFS binding sites from *Zobellia galactanivorans* and *Cellvibrio mixtus* [43, 48] cluster together, (PDB IDs: 5FUI and 1UYY, respectively). These two CBM6s display structural conservation of their binding sites and both interact with chemically similar, mixed-linkage glucans. The emergence of the functional CFS appears to have occurred following divergence from the xylanase associated clade and also contains the CBM6 *Ruminiclostridium thermocellum* ATCC 27405 (PDB ID: 5LA2; [49]). The neoagarooligosaccharide binding CBM6 from *Saccharophagus degradans* 2–40 (PDB ID: 2CDP; [40]) forms the founding member of a clade that is entirely populated by CBMs associated with agarases. In contrast, the binding of xylo- and cellulo-configured ligands is distributed throughout the CBM6 tree, suggesting it may represent the ancestral binding specificity for this family. Similar to what is described above for CAZyme specificities, the integration of structural and functional information into more inclusive trees built with biochemically characterized or uncharacterized sequence datasets helps to improve the accuracy of identifying novel CBM binding specificities in polyspecific families.

To test the accuracy of SACCHARIS to identify novel CBM functions, two genes with CBM6s attached to catalytic modules predicted to digest yeast mannan were targeted for functional characterization. Yeast mannan is an extracellular cell wall polysaccharide found on surface of *Saccharomyces cerevisiae* [19]. It is a mannose rich polysaccharide that contains an extensive α-1,6-mannan backbone decorated by side-chains displaying species-specificity in the linkage chemistry and carbohydrate composition [50]. Deconstruction of a highly complex polysaccharide, such as *S. cerevisiae* mannan, requires the combinatorial action of many different enzymes, including α-mannanases, α-mannosidases, and sugar phosphatases [19]. Although many GH families have been reported to be involved in yeast mannan deconstruction including GH76s and GH92s [19], there are currently no CBMs known to bind this class of polysaccharide. CBM6s associated with a GH76 (BAA75632.1) from *Bacillus circulans* TN31 (BcCBM6) and a GH92 (AFK83609.1) from *Cellulosimicrobium cellulans* were identified and selected for biochemical characterization (Fig. 3a). Interestingly, although BAA75632.1 is a single module, AFK83609.1 contains three tandem CBM6 modules (CcCBM6a, CcCBM6b, and CcCBM6c) that partition together in the CBM6 tree. When analyzed by affinity gel electrophoresis (AGE), BcCBM6, CcCBM6b, or CcCBM6bc did not display any noticeable retardation in the gel, suggesting there is no interaction with intact yeast mannan. However, CcCBM6a on its own or tethered to other CBM6s (CcCBM6ab and CcCBM6abc) displayed a marked change in mobility (Fig. 3b). This represents the first report for a yeast mannan binding CBM. In this regard, SACCHARIS can be performed on other CBMs families associated with polyspecific parent enzymes to identify other unique binding specificities for CBMs.

### Analysis of an entire CAZome

**Fig. 4:**
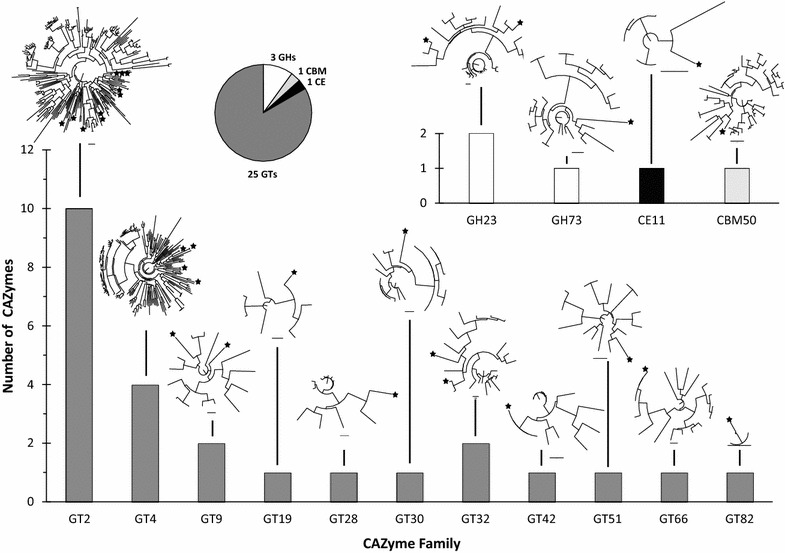
Full CAZome analysis of CAZyme and CBM distributions within a bacterium. Total number of GTs (bottom), and GHs, CEs, and CBMs (top) from *C*. *jejuni* subsp. jejuni NCTC 11168-GSv. Phylogenetic trees generated for each family using *C. jejuni* sequences (black star) embedded into characterized sequence trees. Inset: pie chart of total CAZome sequences extracted from CAZy [1] represented by CAZyme class and CBM. Bar represents a distance of 0.5 for all trees

To determine if SACCHARIS can be applied to entire CAZomes, we have performed an analysis of the *C*. *jejuni* subsp. jejuni NCTC 11168-GSv genome. This microorganism was previously sequenced by our group [30] and possesses three GHs, twenty-five GTs, and one CE and CBM (Fig. 4). Automated extractions were aligned with characterized sequences from each GT family (2, 4, 9, 19, 28, 30, 32, 42, 51, 66, and 82), GH23, GH73, CE11 and CBM50, resulting in the generation of fifteen total phylogenetic trees (Fig. 4). Mapping of the *C*. *jejuni* subsp. jejuni NCTC 11168-GSv enzyme sequences were then performed to indicate relatedness to sequences with previously characterized members. This approach, referred to here as ‘CAZome fingerprinting’, differentiates between the metabolic signatures present within the genomes of individual organisms and identifies uniquely partitioning CAZymes for further analysis.

**Fig. 5:**
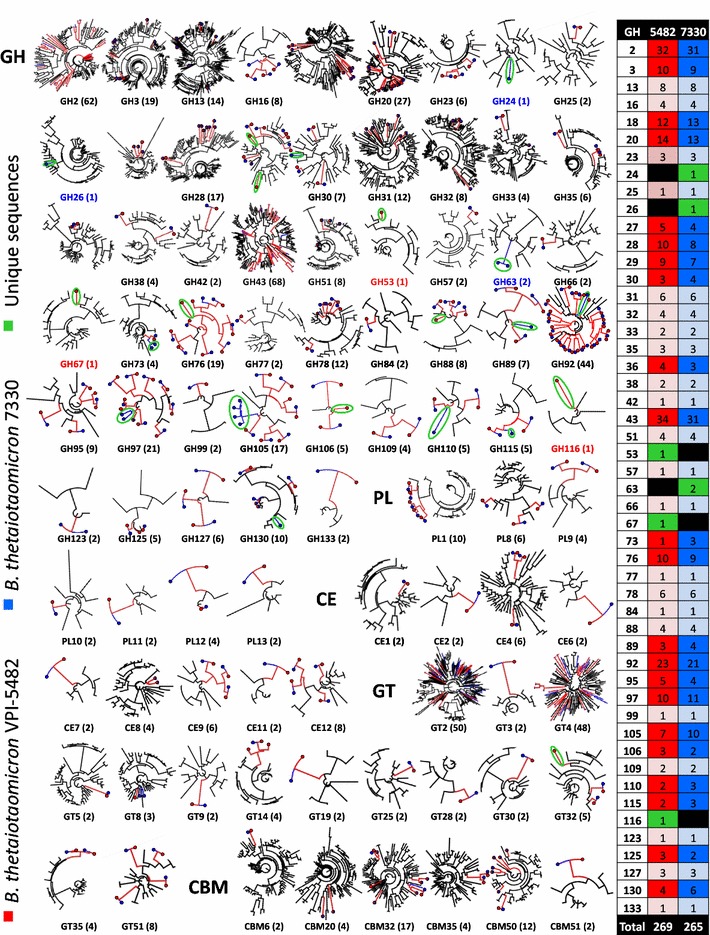
Comparative CAZome fingerprinting for two strains of *B. thetaiotaomicron*. Phylogenetic trees were generated for characterized sequences from every CAZyme and CBM family found in *B. thetaiotaomicron* VPI-5482 (red) and 7330 (blue). Trees are embedded with *B. thetaiotaomicron* sequences for each family and indicated by colour. The total number of entries for each family is listed in parenthesis with exclusive GH family entries for *B. thetaiotaomicron* VPI-5482 and 7330 indicated with red or blue font, respectively. The total number of entries for each strain is listed in a table to the right of the figure. Light shading = identical, dark shading = differential; black = no family entries; green = unique entries. Unique sequences are indicated in the tree with green ellipses

To demonstrate that CAZome fingerprinting can be performed on multiple genomes simultaneously to rapidly compare CAZyme and CBM specificities between related organisms, we have generated CAZome fingerprints for *B. thetaiotaomicron* VPI-5482 (*n* = 269) and *B. thetaiotaomicron* 7330 (*n* = 265), and integrated with characterized sequences (Fig. 5). This setup enables the rapid, visual inspection of every CAZyme and CBM from both strains by providing distance matrixes for sequence relatedness and characterized sequences. In total 86 trees were generated, which include 80 families that are populated by sequences from both strains, and 6 families that contain entries from only one of two strains (e.g., *B. thetaiotaomicron* 7330 has exclusive GH24, GH26, and GH63 sequences; *B. thetaiotaomicron* VPI-5482 has exclusive GH53, GH67, and GH116 sequences). The absence or presence of unique CAZymes in a genome suggests that there are differential relationships between enzyme activities, and potentially, saccharification of unique substrates. Sequences that do not have orthologs in both genomes (indicated by green ellipses) also make promising candidates for enzyme discovery, and may inform functional specificity that exists between two organisms. Tree density is affected by the number of characterized enzymes within the database, and the number of sequences within the genomes of each strain. This also provides a rapid comparison for families that are underpopulated with characterized sequences, which may make candidate families for deeper exploration. Potentially CAZome fingerprinting can be further extended to communities and meta-datasets, which could be informative for forecasting ecosystem responses to different substrates.

## Conclusions

Due to the dynamic nature of genome annotation and deposition, rapid and flexible bioinformatic tools are required to respond to datasets that are increasing in size and complexity. In this regard, SACCHARIS is an automated bioinformatics pipeline tailored for generating phylogenetic trees from CAZyme families. Such analyses have utility for informing function within sequence datasets and streamlining biochemical characterization of targets, such as the first described α-glucanase activity for GH43 presented here. SACCHARIS is also able to generate phylogenies using characterized and structurally defined CBM sequences to streamline the discovery of new binding specificities. For example, we were able to identify the first CBM known to bind yeast mannan. We also demonstrate that SACCHARIS can be applied to entire genomes, creating ‘CAZome fingerprints’ that are useful for differentiating between the saccharolytic specificity of related organisms. Therefore, we believe that SACCHARIS is an in silico tool that will help guide CAZyme and CBM discovery from sequence datasets (Fig. 5).

The modular nature of the SACCHARIS pipeline makes it adaptable for enzyme discovery in protein classes other than CAZymes, such as kinases (e.g., kinase.com; [51]), peptidases (e.g., MEROPS; [52]), and transporters (e.g., Transporter Classification Database; [53]). Inclusion of these databases would require the integration of new HMM (hidden Markov model) profiles with hmmscan to identify catalytic or associated domains in enzyme families based on Pfam or other similar profile annotations. In this regard SACCHARIS represents a useful platform that can be expanded to facilitate enzyme discovery and functional protein characterization in a variety of scientific fields.

## Methods

SACCHARIS incorporates a modularized, tiered approach to extract, identify, prune, align and plot sequences into functional groups. The pipeline design incorporates in-house software and currently available online bioinformatics tools. Local installation of tools such as dbCAN [26], MUltiple Sequence Comparison by Log-Expectation (MUSCLE) [54], ProtTest [55], FastTree [56], and Randomized Axelerated Maximum Likelihood (RAxML) [57] are required for proper functionality. SACCHARIS has been installed and run on an Intel-i7 laptop with 32 GB of RAM and a 48-core server with 1 TB of RAM, both running Debian Linux (http://www.debian.org/). Modularity has been built into the pipeline to facilitate simplified exchange of bioinformatic tools, such as using FastTree in place of RAxML, thereby diversifying its utility. This design requires the creation of transition scripts for input and output files to enable seamless flow between modules. The open-source nature of this architecture also affords the user the freedom to tailor data analysis for project-specific applications and enables the pipeline to be integrated with large-scale bioinformatic work-flow environments.

### Sequence retrieval

CAZy sequences from selected families are extracted with an in-house program. User input of family, family number and grouping are used to extract accession numbers remotely from the CAZy database. For GH43: 144 characterized sequences and 7136 uncharacterized sequences were extracted, and 6 and 443 entries were removed, respectively, to account for duplications and fragments. For CBM6: ninety-nine sequences were extracted and ninety sequences were used.

- Examples of user input include:
- ‘–f GH43 –g “all,characterized”’
- Or
- ‘–f CBM6 –g characterized’,

where the ‘GH’ glycoside hydrolase or ‘CBM’ carbohydrate binding module are the family, and ‘43’ or ‘6’ are the family number and, ‘all’ and ‘characterized’ the grouping. By selecting and sorting with accession numbers, NCBI [58] can be accessed for retrieval of protein sequence data.

The retrieval code is tailored for the current design of the CAZy website. Alterations may be required if there are future modifications to its structure and/or design. CAZy currently lacks an application program interface (API). As such code developed to extract data from CAZy relies on the ability to download and parse HTML source. Additional features have been built into the code to address functional errors that were discovered during extraction of some family datasets. Examples include; eliminating duplicate sequences resulting from the presence of multiple accession numbers, removing sequence annotated in CAZy as fragments, linking sequences from multiple webpages (i.e., currently a maximum of 1000 entries from a CAZy family are displayed per page), retrieving sequence data for samples with no accession number, and deciphering actual accession numbers from accession-like numbers in the description field.

Accession numbers extracted from CAZy are used to create a URL submission to NCBI through the e-search function of the Entrez API. 

Results of entrez e-search are then used to create a second URL e-fetch submission. 

The return of the e-fetch submission is protein sequence data in FASTA format. The Entrez API utility within NCBI is limited to 500 requests per submission so larger families need to be split into a linked series by creating temporary FASTA files that are merged upon completion of the code.

Accession numbers are not included in the ‘structure’ grouping on CAZy, therefore, the ability to extract protein sequence data is performed via a focus shift. When the grouping of ‘structure’ is detected by the code the extraction application switches from accession number detection to protein data bank (PDB) identifier detection. Protein sequence data is extracted from the PDB website ([59]; http://www.rcsb.org/pdb/home/home.do) using the list of PDB identifiers. The PDB website, unlike NCBI does not have an e-search function for sequence retrieval; therefore, the PDB identifier was used create a URL and through the use of a HTTP get protocol extract the matching protein sequence data in FASTA format.

### Pruning of full length sequences to CAZyme modules

Extracted datasets can be augmented with additional sequences prior to CAZyme module identification by SACCHARIS; sequences must be entered in FASTA format. All sequences are jointly run through dbCAN [26] to identify modular boundaries. Outputs from dbCAN are scanned to retrieve sequence identifiers and start and stop locations of the sequences with hits matching the user family selection.

Sequence identifiers are then used by the program to extract only those sequences from the combined CAZy-User dataset and the sequence data is pruned at both the N-terminus and C-terminus of the proteins. Provisions are built into the program for special cases when a sequence is multimodular (i.e., contains more than one copy of the enzyme or CBM module). Modules from multimodular proteins are treated individually, and exported with the delineation ‘_#”, where ‘#’ equals the sequential position of the tandem module in alphabetical order (e.g., _A, _B, etc.). The subsequent pruned FASTA file is collated and used for entry into the alignment module.

### Aligning of sequences

MUSCLE identifies each sequence through the first 10 characters of the sequence identifier of the inputted FASTA file which will hamper downstream processing if there are identical alignment identifiers [54]. Therefore, a script that runs prior to the incorporation of user-generated sequences was developed to create a unique 10 character identifier for each sequence. This identifier is placed at the beginning of each sequence. At present it is recommended a unique 10 character identifier starting with ‘U’ and followed by nine digits be added to additional user sequences prior to being added to the dataset.

### Plotting of aligned sequences

For RAxML or FastTree to generate optimal trees it is recommended a best-fit model be selected. Best-fit model selection is performed using ProtTest [55]. ProtTest output is redirected to a file which is then parsed utilizing code built into SACCHARIS. The output file writes to an array. The best model is selected based upon the results of the ProtTest scoring matrix for each of the tests run by ProtTest and a best-fit model is selected. The final step of the code uses the model selection to create either a FastTree or RAxML input string for the model depending on which plotting program was selected by the user. For example, if ProtTest identified JTT-IG the subsequent input for RAxML would be PROTGAMMAIJTT and for FastTree would be gamma-jtt. 

Notably, ProtTest is limited to 4000 sequences (i.e., taxa); therefore, for datasets exceeding 4000 sequences SACCHARIS performs a randomized selection of 1500 sequences for the muscle alignment to approximate phylogenetic distances. Randomized selection is performed using fasta_subsample.pl script [60] against extracted CAZy sequences. The randomized subsample is run through dbCAN, pruned and aligned using MUSCLE. Alignment outputs are analyzed by ProtTest to select the best-fit model for tree building. During development, the accuracy of randomized selection was demonstrated by performing repeated random analysis of the same GH43 dataset (*n* = 6), which resulted in the selection of the same model with an accuracy of 0.83.

Phylogenetic analysis proceeds using best-fit MUSCLE aligned data with the user selection of FastTree or RAxML. When selecting a default program, it is important to consider efficiency. Calculation of bootstrap values is computationally intensive and thorough. Therefore, when you have a large alignment file (> 1000 taxa) calculating bootstrap values can take a significant amount of time. FastTree uses a pseudo-bootstrap (local support values) calculation with the Shimodaira–Hasegawa test [61], thereby eliminating the computationally intensive part and producing a result very quickly. With RAxML, users may define the number of bootstrapping iterations or allow RAxML to determine the optimal number of iterations. For SACCHARIS we have instituted a threshold of 100 bootstrap iterations when RAxML is selected. Total bootstrap iterations well below a 100 can take hours and iterations over 100, days to weeks, depending on the system. Time to complete is dependent on number of aligned sequences and CPU architecture.

Removing duplicate copies of entries helps to generate optimal runs and prevent run crashes. In the case that RAxML identifies identical sequences with unique identifiers a reduced alignment file with the identical sequences removed will be created. The end output is a Newick format file for phylogenetic trees. FigTree (http://tree.bio.ed.ac.uk) was used to generate trees, and sequences of interest were manually highlighted.

For CAZome extractions, SACCHARIS can be run sequentially or in parallel via user made scripts so as to extract each CAZy family identified. Genome sequences identified as part of the CAZome can be added to each call by SACCHARIS thereby creating CAZyme family ‘fingerprints’.

### Purification and characterization of CBM6 modules

Codon optimized gene sequences corresponding to amino acid residues 385–525 of *B. circulans* TN31 Aman6 (GenBank Accession Number: BAA75632.1) and residues 970–1440 of *C. cellulans* Man5 (GenBank Accession Number: AFK83609.1) were synthesized (BioBasic) and subcloned into pET28a to create the pET28-BcCBM6 and pET28-CcCBM6abc plasmids, respectively. Nucleotide sequence corresponding to residues 976–1280, 1127–1427, 976–1120, 1127–1280, and 1281–1427 were subcloned into the NdeI and XhoI sites of pET28a to generate pET28-CcCBM6ab, pET28-CcCBM6bc, pET28-CcCBM6a, pET28-CcCBM6b, and pET28-CcCBM6c. Constructs were transformed into *E. coli* BL21 Star (DE3) cells and grown at 37 °C to an OD 600 nm of 0.8 in LB broth containing kanamycin (50 µg ml^−1^). Gene expression was induced with 0.22 mM IPTG at 16 °C overnight. Cells were harvested by centrifugation and lysed in 20 mM Tris pH 8.0, 500 mM NaCl by sonication. Lysates were cleared by centrifugation and loaded onto Ni–NTA resin for purification by immobilized metal affinity chromatography. Recombinant BcCBM6, CcCBM6a, CcCBM6b, CcCBM6ab, and CcCBM6abc were eluted in a linear gradient of imidazole and fractions containing significant amounts of pure protein as judged by SDS-PAGE were pooled and buffer exchanged into 20 mM Tris pH 8.0. Protein concentrations were determined using the Beer-Lambert law with estimated extinction coefficients of 19,940, 99,350, 62,910, 64,400, 33,460, and 29,450 M^−1^ cm^−1^ for BcCBM6, CcCBM6abc, CcCBM6ab, CcCBM6bc, CcCBM6a, and CcCBM6b, respectively [62].

AGE was performed as described previously [63], with the following modifications. Native polyacrylamide gels (10% acrylamide, 25 mM Tris pH 8.8, 250 mM glycine) were prepared with and without the addition of 1% yeast mannan (Sigma #M3640). 3 µg of Bovine Serum Albumin, BcCBM6, CcCBM6a, CcCBM6ab, CcCBM6bc, and CcCBM6abc were loaded on gels and separated in native running buffer (25 mM Tris pH 8.3, 193 mM glycine) at 110 V for 3 h at 4 °C. Protein migration was visualized by staining with Coomassie blue.

### Purification and characterization of BdGH43b

Codon optimized gene sequences GH43b (BdGH43b) corresponding to amino acid residues 317–624 of WP_007831524.1 was synthesized (BioBasic) and subcloned into pET28a to generate a C-terminal poly-histidine tagged fusion of the protein. Constructs were transformed into *E. coli* BL21 Star (DE3) cells. Positive transformants were grown at 23 °C to an OD 600 nm of 0.5 and induced with 0.05 mM IPTG for 4 h. Inclusion bodies containing the recombinant protein were prepared as described [64] and further purified by centrifugation in 2 M sucrose. Harvests were extracted for 10 min in 25 mM Tris (pH 8), 200 mM NaCl, and 8 M urea with shaking at 30 °C. This extraction protocol leaves much of the irreversibly modified proteins in the inclusion bodies, which are pelleted after centrifugation at 12,000*g* for 10 min. Solubilized proteins were fractionated by adsorption on a nickel column in 8 M urea, 0.5 M NaCl. To refold, bound proteins were washed step wise in 4 column volumes of 6, 4, 2 M urea at a flow rate of < 0.1 ml min^−1^ and eluted in a 2 M urea buffer containing 300 mM imidazole. Eluates were dialyzed overnight in 25 mM Tris (pH 8), 200 mM NaCl, 5 mM β-mercaptoethanol. Protein fractionation and determination of pure protein was performed by SDS-PAGE. Concentration was calculated using an estimated extinction coefficient of 74,260 M^−1^ [62]. Enzymatic activity of purified BdGH43b (2 μM) was assayed using 0.5 mg ml^−1^ water soluble starch (Sigma-Aldrich S9765) in 30 mM potassium phosphate buffer (pH 5.8) at 37 °C overnight. When indicated, reactions were terminated by heating at 100 °C, supplemented with 10 U of α or β-amylase (Sigma-Aldrich A3403, A7130), and incubated at 37 °C for an additional 30 min. After heat inactivation, reactions were spun at 14,000*g* for 2 min, resolved by TLC on silica gel matrix (EMD Millipore 105553), and visualized using orcinol staining, or by fluorophore-assisted carbohydrate electrophoresis as described in [65].

## Abbreviations

AGE: affinity gel electrophoresis
API: application program interface
AAs: auxiliary activities
CAZyme: carbohydrate active enzyme
CBM: carbohydrate binding module
CE: carbohydrate esterase
CFS: concave face site
dbCAN: database for automated Carbohydrate-active enzyme ANnotation
GH: glycoside hydrolase
GH43: glycoside hydrolase family 43
GH92: glycoside hydrolase family 92
GT: glycosyl transferase
MUSCLE: Multiple Sequence Comparison by Log-Expectation
PL: polysaccharide lyase
PULDB: Polysaccharide Utilization Loci Database
PDB: protein data bank
RAxML: Randomized Axelerated Maximum Likelihood
SACCHARIS: Sequence Analysis and Clustering of Carbohydrate Active enzymes for Rapid Informed prediction of Specificity
VLS: variable loop site
